# Electrocardiographic criteria for silent myocardial infarction: Impact of different definitions on detection rates and prognostic significance in the atherosclerosis risk in communities (ARIC) study: A comparison with evolving bundle branch blocks

**DOI:** 10.1016/j.jelectrocard.2025.154174

**Published:** 2025-12-06

**Authors:** R. Brandon Stacey, Ronald J. Prineas, Zhu-Ming Zhang, Bruce M. Psaty, Wayne Rosamond, Lynne Wagenknecht, Elsayed Z. Soliman

**Affiliations:** a Section on Cardiovascular Medicine, Department of Medicine, Wake Forest School of Medicine, Winston-Salem, NC, United States of America; b Division of Public Health, Wake Forest School of Medicine, Winston-Salem, NC, United States of America; c School of Public Health, University of Washington; Seattle, WA, United States of America; d Gillings School of Public Health, University of North Carolina; Chapel Hill, NC, United States of America

**Keywords:** Myocardial infarction, Electrocardiography, Silent myocardial infarction

## Abstract

**Introduction:**

It is unclear how differences in the electrocardiographic (ECG) definition of myocardial infarction (MI) impact detection rates and prognostic significance of silent MI (SMI).

**Methods:**

This analysis included 9188 participants (57.4 % women, 20 % black, age 62.6 ± 6.0 years) enrolled in the Atherosclerosis Risk in Communities study who had serial ECGs obtained between visit 1 (1987–1989) and visit 4 (1996–1998). Exclusions included known cardiovascular disease (CVD) prior to visit 1, ECG findings of MI or bundle branch block (BBB) at visit 1, or an adjudicated fatal and non-fatal MI events between visits 1 and 4. Using the Minnesota Code (MC) ECG Classification and in the absence of adjudicated MI, the following SMI definitions were derived: Standard MC MI [major Q-wave abnormality, or minor Q-wave abnormality plus major ST/T abnormality], only major Q-wave abnormality, standard significant serial Q-wave changes [Q1 to Q7], expanded MC serial Q-wave changes[Q1 to Q8], standard MC significant serial Q-wave changes or Standard MC significant serial ST/T changes, and evolving MC BBB. Cox proportional hazard models were used to examine the association of different definitions of SMI (compared to no new MI or evolving BBB) with fatal or non-fatal MI events ascertained after visit 4 until December 2016.

**Results:**

The prevalence of SMI ranged from 0.6 % to 7.0 % depending on the ECG criteria defining SMI. Presence of SMI was predictive of fatal/non-fatal MI regardless of the definition but with varying levels of association. Standard MC expanded serial Q-wave changes had the strongest adjusted relationship [Hazard Ratio: 2.53 (95 % Confidence Interval (CI): 1.60–4.01)] while evolving BBB had the weakest adjusted association [HR: 1.39 (95 % CI: 0.80–2.40)].

**Conclusions:**

The prevalence and prognostic significance of SMI are impacted by the ECG criteria defining MI. A uniform approach(s) for detection of SMI in population studies which builds on the available standard definitions that fit different research scenarios is needed.

## Introduction

Despite significant gains in preventative health, coronary heart disease continues to be a leading cause of morbidity and mortality world-wide [1]. In clinical practice, symptoms typically drive the assessment and management of the patients [2–4]. However, a significant proportion of individuals who experience a myocardial infarction (MI) may experience only mild or no symptoms [5,6]. In various cohort studies, silent (or unrecognized) MIs account for one-third to nearly one-half of MIs seen during follow-up [7–13]. Individuals who experience a silent MI are more likely to be under-treated and may be exposed to increased risk for subsequent coronary events, heart failure, stroke, and sudden death [14–22].

While electrocardiogram (ECG) remains one of the most used tools to diagnose silent MIs, little data has been used to directly compare different definitions for potential silent MI on ECG in population studies. Part of the challenge in identifying those with silent MIs rests upon our ability to successfully identify them, and clinicians’ ability to do this may be significantly limited by not knowing the best definition to use [23–25]. Both echocardiography and MRI can directly visualize the evidence of a prior silent MI, but they are not universally available, and in many instances, may be cost-prohibitive, particularly at the public health level [26,27].

To determine the best criteria to recognize a prior silent MI on ECG, we turned to the Atherosclerosis Risk in Communities (ARIC) cohort study. By optimizing our understanding of the different definitions available to diagnose a prior MI, this knowledge may be used to better identify those who have experienced a silent MI, and consequently, they may receive the appropriate care and intervention needed to reduce their risk of subsequent clinical events and mortality.

## Methods

### Study population

The ARIC study was designed to investigate the causes of atherosclerosis and its clinical outcomes, as well as variation in cardiovascular risk factors, medical care, and disease by race and sex [28]. From 1987 to 1989 (ARIC study visit 1), 15,792 adults (55.2 % women; age, 45–64 years) from 4 US communities (Washington County, Maryland; suburbs of Minneapolis, Minnesota; Jackson, Mississippi; and Forsyth County, North Carolina) were enrolled and underwent a phone interview and clinic visit. Additional examinations were conducted in 1990–1992 (visit 2), 1993–1995 (visit 3), and 1996–1998 (visit 4) with the most recent being visit 11 which started in March 2024. Participants were mostly white in the Washington County and Minneapolis sites, exclusively black in Jackson, and a mix of both in Forsyth County. The study was approved by each study site’s institutional review board. All participants provided written informed consent.

For the purpose of this analysis, we included all ARIC participants with good quality and complete ECG data at visits 1 to 4 as well as outcome events after visit 4. Visit 4 served as our baseline exam for longitudinal analyses. We excluded the following participants: 136 with poor quality ECG, 3775 with missing ECG in any of the ARIC first 4 visits (including 871 who died before visit 4), 429 with ECG diagnosis of external pacemaker or Wolff-Parkinson-White pattern, and 277 with missing one or more of baseline cardiovascular disease (CVD) risk factors. We also excluded 1706 participants with a history of CVD at baseline which was defined as the presence of ECG evidence of MI or a self-reported history of physician-diagnosed MI, coronary artery bypass surgery, coronary angioplasty, heart failure, or stroke. A further 386 were excluded who experienced a clinically-recognized MI between visit 1 and visit 4. After all exclusions, 9188 remained and were included in the analysis.

### Silent MI

SMI was defined as ECG evidence of new MI/evolving bundle branch block at ARIC visit 2, visit 3 or visit 4 that was not present at visit 1 in the absence of documented CMI. Participants with both SMI and CMI between ARIC visit 1 and visit 4 were considered as having CMI and excluded. Identical electrocardiographs (MAC PC, Marquette Electronics Inc., Milwaukee, Wisconsin) were used at all clinical sites, and resting 10-s standard simultaneous 12-lead ECGs were recorded in all participants using strictly standardized procedures. All ECGs were processed in a central ECG laboratory (initially at Dalhousie University, Halifax, Nova Scotia, Canada and later at the EPICARE Center, Wake Forest School of Medicine, Winston-Salem, North Carolina, USA), where all ECGs were visually inspected for technical errors and quality.

ECG evidence of MI was defined by new appearance of Minnesota Code ECG Classifications as the any of the following criteria used separately:[29].

Standard MC MI: a major Q/QS wave abnormality (MC 1.1.1, 1.1.2, or 1.1.5 or MC 1.2.1, 1.2.2, or 1.2.5), or minor Q/QS waves abnormality (MC 1.3.1, 1.3.2, or 1.3.5) plus major ST-T abnormality (MC 4.1, MC 4.2, MC 5.1 or MC 5.2);

Only major Q-wave abnormality (MC 1.1.1, 1.1.2, or 1.1.5; 1.2.1, 1.2.2, or 1.2.5);

Standard significant serial Q-wave changes (MC serial Q1-Q7);

Expanded MC serial Q-wave changes (MC serial Q1-Q8);

Standard significant serial Q wave changes or standard significant serial ST/T changes (MC serial Q1-Q7 or MC serial ST1-ST8);

Evolving BBB (MC EBBB1-EBBB3).

### Fatal and non-fatal Clinical MI

MI deaths and hospitalizations were ascertained after ARIC visit 4 (1996–1998) through December 2016. These events were reviewed in each clinical center during an annual follow-up phone interview or through review of community hospital discharge indexes. Incident MI events included definite or probable hospitalized MI (clinical MI, i.e. CMI in this analysis) or definite MI death. All MI events classification and specific criteria including the adjudication process have been previously described [30,31].

### Covariates

Baseline age, sex, race, education level, income, and smoking status were determined by self-report. Body mass index at baseline was calculated as weight in kilograms divided by height in meters squared. Blood samples were obtained after an 8-h fasting period. Baseline diabetes mellitus was defined as a fasting glucose level ≥ 126 mg/dL (or nonfasting glucose ≥200 mg/dL), a self-reported physician diagnosis of diabetes mellitus, or the use of diabetes medications. Baseline hypertension was defined as systolic blood pressure ≥ 140 mmHg, diastolic blood pressure ≥ 90 mmHg, or the use of blood pressure–lowering medications. At each study visit, medication history was obtained by self-report of medication intake during last 2 weeks and by reviewing medications brought by the participants to their visits. Each medication was coded by trained and certified interviewers with the use of a computerized medication classification system.

### Statistical methods

Frequency distributions of the variables used in analyses were first inspected to rule out anomalies and outliers. Descriptive statistics were used to determine mean values, standard deviations, and percentile distributions for continuous variables, and frequencies and percentages for categorical variables.

### Kaplan-Meier curves were used to evaluate survival free from fatal/non-fatal MI

Cox proportional hazards analysis was used to examine the associations of SMI occurring from visit 1 to visit 4 with fatal and non-fatal MI occurring after visit 4. The follow up time included the time from visit 4 to the event. Non-MI deaths were treated as censored. Models were incrementally adjusted as follows: Model 1 adjusted for baseline demographics (age, sex and race), and Model 2 adjusted for variables in Model 1 plus study field center, body mass index, smoking status, systolic blood pressure, blood pressure lowering medications, diabetes mellitus, total cholesterol, use of cholesterol lowering medications (all variables measured at baseline).

All analyses were performed with SAS version 9.3 (SAS Institute Inc., Cary, North Carolina). A 2-sided *p* < 0.05 was considered significant.

## Results

This analysis included 9188 participants (age at 4th ARIC visit was 62.6 (± 5.7) years, 56.9 % women, and 20.3 % African American). From baseline data obtained from the 4th ARIC visit, the characteristics of the participants with versus without different ECG-MI criteria did not differ by method (Table 1). Overall, those who met criteria tended to be older, male, and experienced numerous traditional cardiovascular risk factors.

The prevalence of SMI was highest for the combined serial Q wave changes or serial ST/T changes (7 %; Table 2). The prevalence of SMI by major Q wave or minor q wave with ST/T changes was 3.9 %. The lowest prevalence of SMI was with serial Q wave changes (0.6 %; Table 2).

**Fig. 1.**
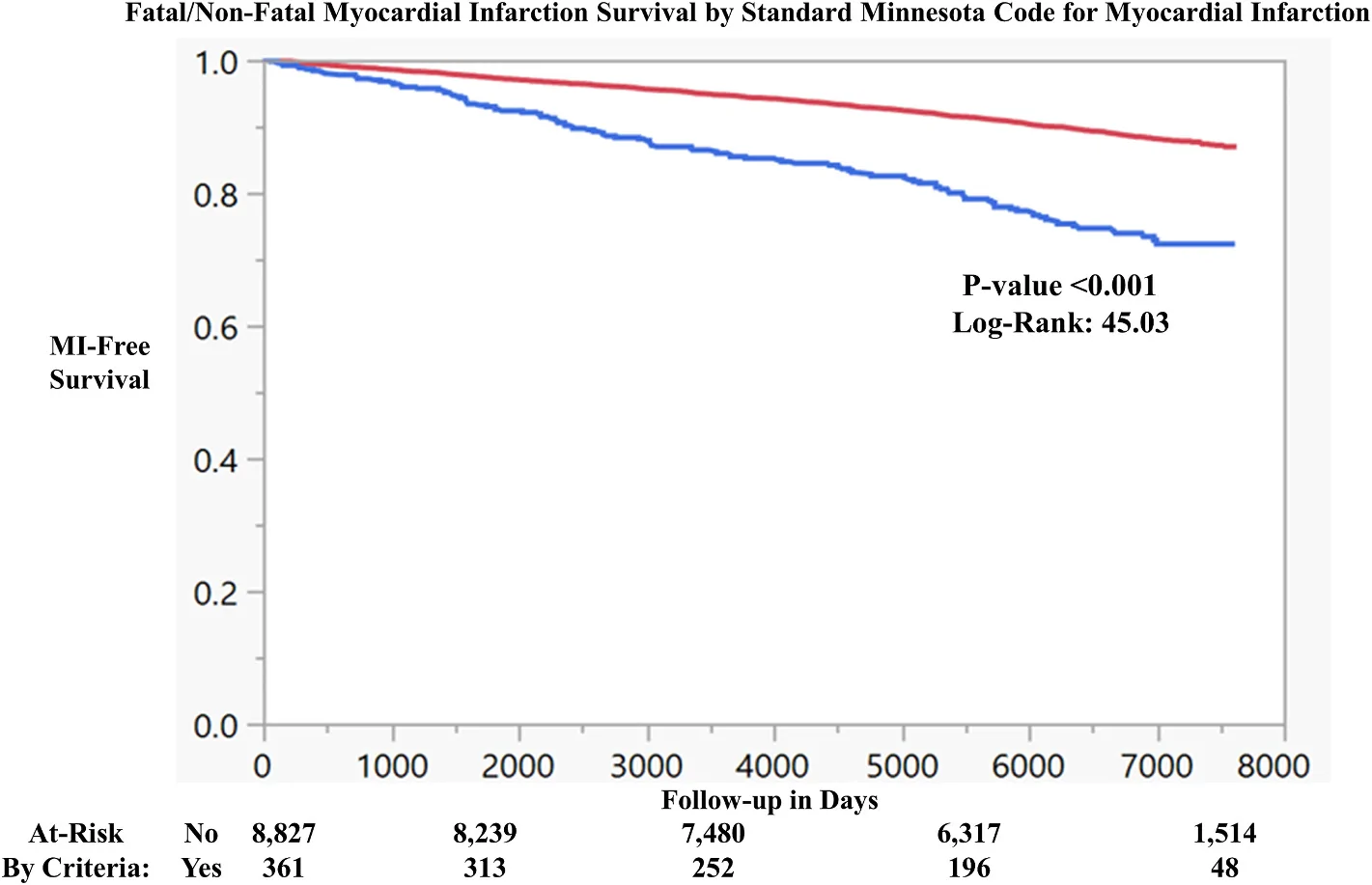
Kaplan-Meier Curve for fatal/non-fatal MI by Standard MC (Red-normal; Blue-criteria met). (For interpretation of the references to colour in this figure legend, the reader is referred to the web version of this article.)

**Fig. 2.**
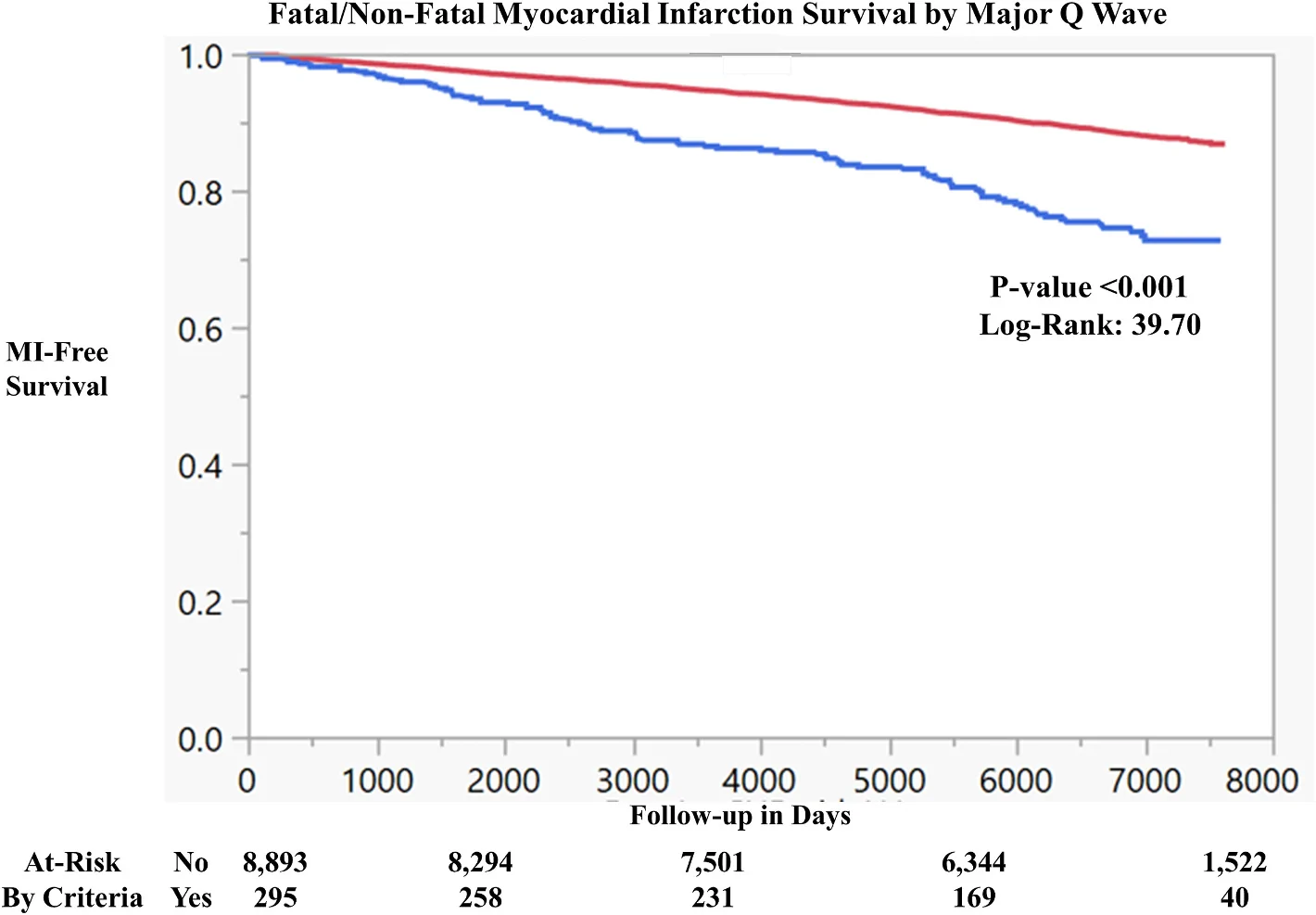
Kaplan-Meier Curve for fatal/non-fatal MI by Major Q wave (Red-normal; Blue-criteria met). (For interpretation of the references to colour in this figure legend, the reader is referred to the web version of this article.)

**Fig. 3.**
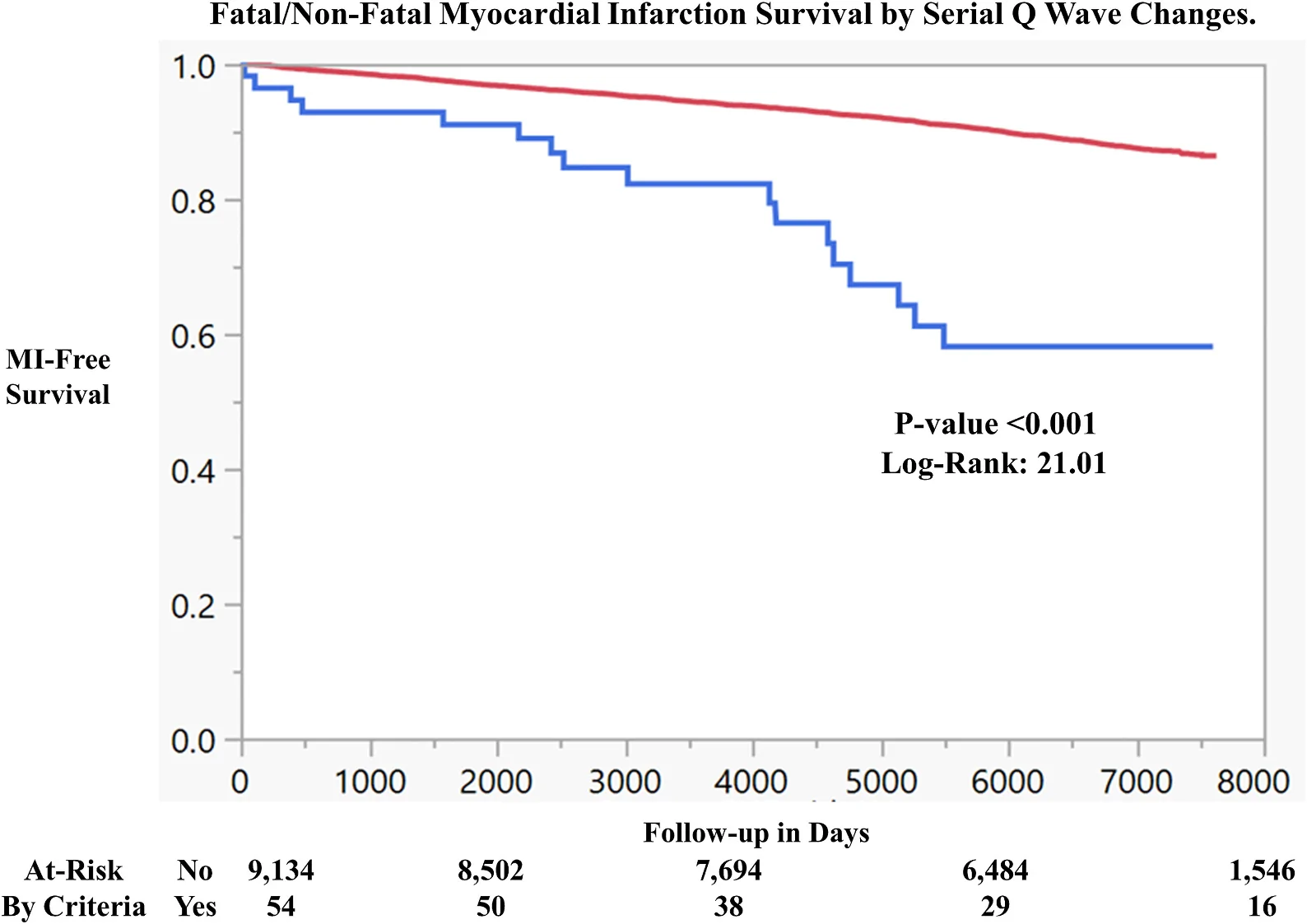
Kaplan-Meier Curve for fatal/non-fatal MI by serial Q wave changes. (Red-normal; Blue-criteria met). (For interpretation of the references to colour in this figure legend, the reader is referred to the web version of this article.)

**Fig. 4.**
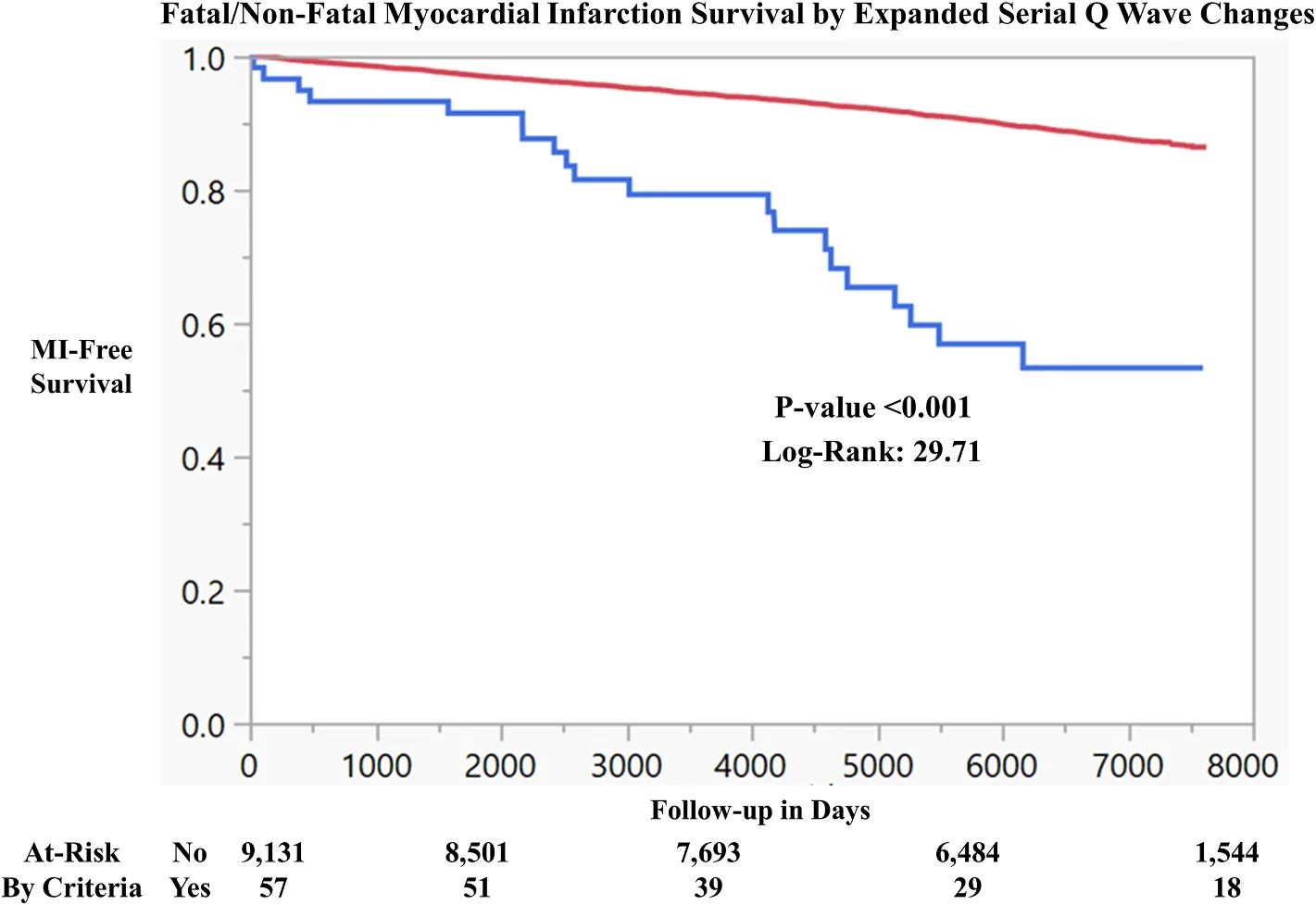
Kaplan-Meier Curve for fatal/non-fatal MI by expanded serial Q wave changes. (Red-normal; Blue-criteria met). (For interpretation of the references to colour in this figure legend, the reader is referred to the web version of this article.)

**Fig. 5.**
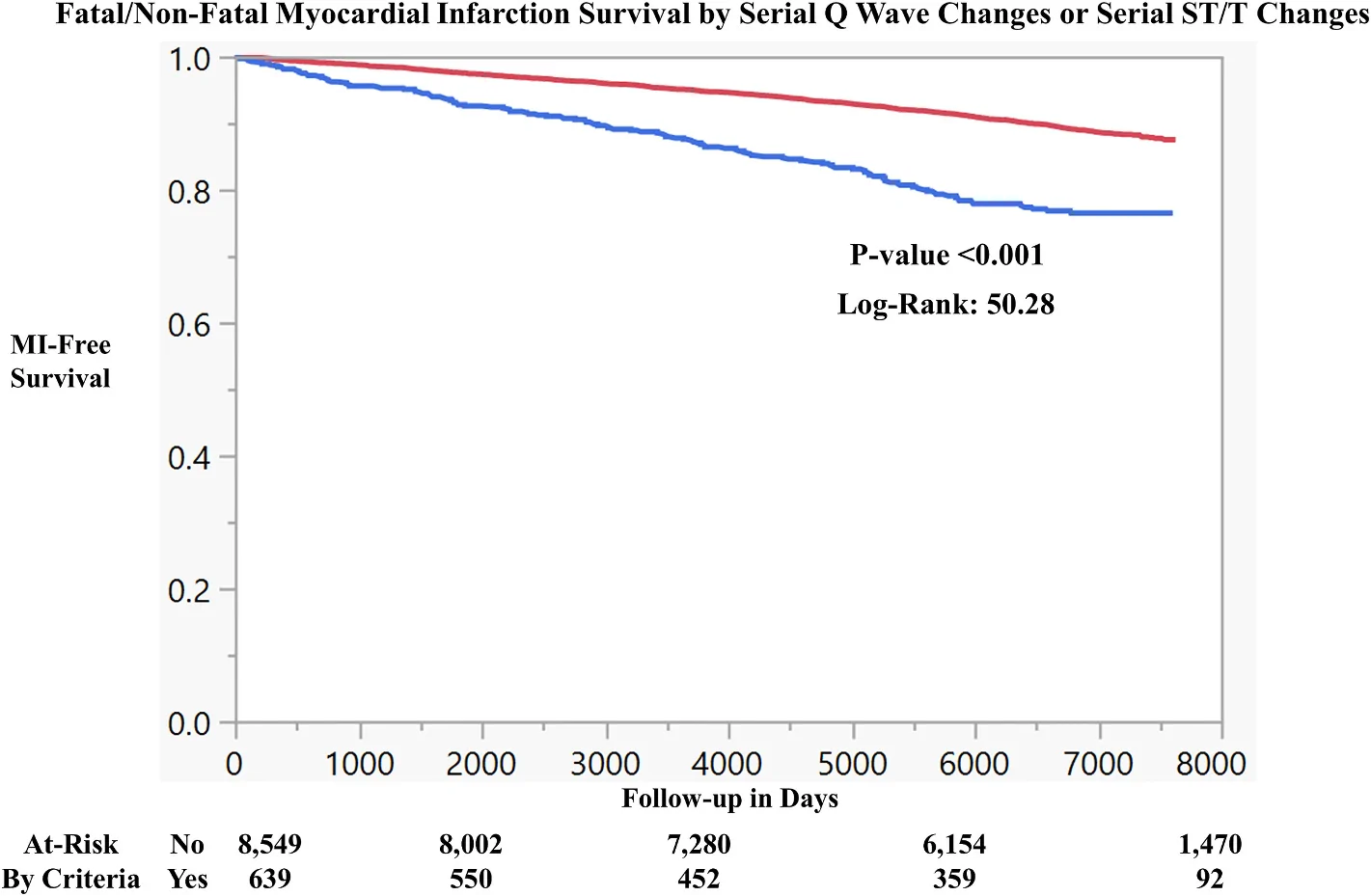
Kaplan-Meier Curve for fatal/non-fatal MI by serial Q wave changes or serial ST/T changes. (Red-normal; Blue-criteria met). (For interpretation of the references to colour in this figure legend, the reader is referred to the web version of this article.)

**Fig. 6.**
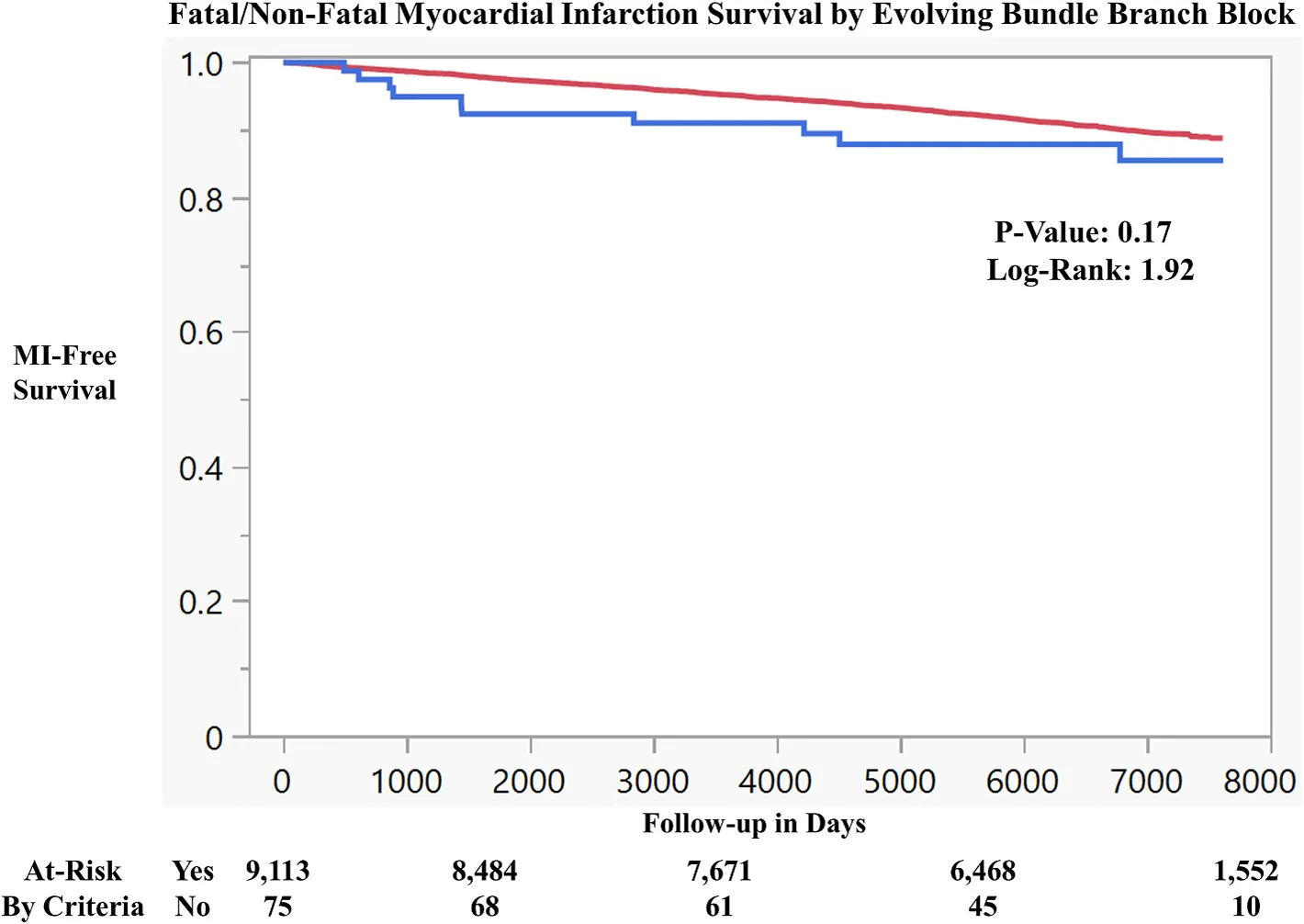
Kaplan-Meier Curve for fatal/non-fatal MI by evolving BBB. (Red-normal; Blue-criteria met). (For interpretation of the references to colour in this figure legend, the reader is referred to the web version of this article.)

All ECG criteria were significantly associated with less survival free from fatal/non-fatal MI, but evolving BBB had the weakest association (Figs. 1–6).

In Cox proportional hazard models adjusted for demographics, all ECG criteria except evolving BBB were associated with fatal/non-fatal MI, with serial change either standard or expanded having the strongest associations. The same patterns persisted after further adjustment for cardiovascular risk factors (Table 2).

## Discussion

In this analysis from the ARIC study, one of the largest community-based cohort studies in the US, we examined the differences in the incidence and prognosis of silent MI by comparing commonly used criteria to evaluate their relationship to clinical outcomes. The three key findings are: 1) The definition used may significantly impact our ability to identify those with a possible silent MI; 2) Those with serial Q wave changes experienced the highest risk for fatal or nonfatal MIs during follow-up; 3) Those with evolving bundle branch blocks encountered the least amount of risk among the different criteria. These findings highlight the challenges and importance of using different ECG-MI criteria in detecting silent MIs and the potential impact of such detection on personalized prevention of coronary events. Failing to use the most appropriate criteria means that those with silent MIs will continue to be deprived from appropriate medical attention compared to those who experience a clinically recognized MI.

The ideal ECG criteria for MI should exhibit the strongest association with subsequent coronary events. Having experienced an MI carries the highest risk for experiencing a subsequent coronary event, and hence, the ECG MI criteria with the strongest association for subsequent coronary events most likely represents a true MI phenotype. The ECG-MI definition significantly impacts the incidence of possible silent MI. Serial changes in Q wave or ST segment identified more individuals than other criteria and experienced similar outcomes relative to the standard ECG-MI definition. The presence of Q waves suggests loss of myocardium, but by including ST segment changes without Q wave changes, this criterion may include those who are experiencing silent ischemia but have not yet experienced a MI [32,33]. Since these individuals are asymptomatic, they may be at higher risk of coronary events due to under-utilization of guideline-directed medical therapy [34]. Consequently, this criterion may be an important tool to identify cardiovascular risk but may over-estimate silent MIs relative to the standard ECG-MI definition. These findings invite further studies to ensure clinical and ECG definitions precisely identify silent MIs compared to those with silent myocardial ischemia and rightly ascribe to each its risk of future coronary events.

Those with an evolving Q wave experienced the highest risk for fatal or nonfatal coronary events during follow-up and may have a higher specificity than other criteria, but this comes at the expense of not identifying a significant amount of participants who did experience a coronary event. Ontologically, an evolving Q wave may represent the most pure definition of an ECG-MI, but many MIs do not result in pathological Q waves on ECG.35 Consequently, this definition may under-identify those who have a silent MI.

Those with evolving bundle branch blocks encountered the least amount of risk among the different criteria. Historically, left bundle branch blocks represented an ST-segment equivalent in appropriate clinical situations. This may have represented a late presentation with significant injury to the proximal septum and the left bundle. Frequently, those who did present with left bundle branch block during a STEMI were in cardiogenic shock. However, as the proportion of MIs shifts from ST-segment elevation to non-ST segment elevation, this may reduce the incidence of those who present with left bundle branch block as the ECG manifestation of a MI. Today, evolving bundle branch blocks most likely reflects conduction system disease as a result of aging. Hence, in the present climate, evolving bundle branch blocks may not be the best criteria to identify silent MIs.

These findings invite several next steps for future directions. Each of these criteria would benefit from a more direct comparison with other validated measures of establishing the presence of MI. Cardiac magnetic resonance imaging remains the gold standard for definitively visualizing scar formation from MIs with late gadolinium enhancement. While late gadolinium enhancement can visualize an infarct, pattern recognition must be used as fibrosis and inflammation involves the mid-myocardium and the epicardium, but MIs nearly always involve the endocardium. Cardiac CT may also represent a potential pathway to confirm a significant coronary event by visualizing obstructive coronary disease. Both of these methods directly visualize either scar tissue or occluded coronary arteries. However, both methods are not universally available, and in many instances, they are cost prohibitive.

### Limitations

As with all studies, there are limitations which must be considered in interpreting these findings. First, ECG may under-estimate the true incidence of SMI [35]. In the Multi-Ethnic Study of Atherosclerosis, almost 8 % of participants had evidence of myocardial scar on MRI using late gadolinium enhancement, but ECG only identified 22 % of these [36]. In reality, the number of participants who experienced a silent MI may be much higher than what we have observed. Some participants may have experienced a silent MI prior to an adjudicated MI between visits 1 and 4, which may under-estimate the true incidence of silent MI. Next, the study’s ability to capture clinical events depended heavily on reviewing documentation to adjudicate such events. Conceivably, not all events may have been reported to the study center, which may slightly under-estimate the true prognosis of having experienced a silent MI. Lastly, for participants who developed ECG criteria for MI, this finding may have influenced their clinical course by encouraging providers to be more sensitive in testing for possible coronary events.

## Conclusion

The prevalence and prognostic significance of SMI are impacted by the ECG criteria defining MI. Criteria based on major Q wave or minor q wave with ST changes aligned best with anticipated clinical outcomes. A uniform approach(s) for detection of SMI in population studies which builds on the available standard definitions that fits different research scenarios is needed.

## Figures and Tables

**Table 1:** Baseline characteristics by ECG criteria.

	Major Q wave abnormality (MC 1.1 or MC 1.2) or minor Q wave abnormality (MC 1.3) plus major ST/T abnormalities (MC 4.1, MC 4.2, MC 5.1 or MC 5.2)		Only Major Q wave abnormality (MC 1.1.x, 1.2.x)		Standard significant serial Q wave changes (MC serial Q1-Q7)		Expanded serial Q wave changes (MC serial Q1-Q8)		Standard significant serial Q (MC serial Q1-Q7) wave changes or Standard significant serial ST/T changes (MC serial ST1-ST8)		Evolving BBB (MC EBBB1-EBBB3)	
						
	Yes (*n* = 361)	No (*n* = 8827)	Yes (*n* = 295)	No (*n* = 8893)	Yes (*n* = 54)	No (*n* = 9134)	Yes (*n* = 57)	No (*n* = 9131)	Yes (*n* = 639)	No (*n* = 8549)	Yes (*n* = 75)	No (*n* = 9113)

Age	64.1 ± 5.8	62.6 ± 5.6*	64.1 ± 5.7	62.6 ± 5.6*	65.0 ± 5.7	62.7 ± 5.6*	65.1 ± 5.6	62.7 ± 5.6*	63.8 ± 5.9	62.5 ± 5.6*	65.1 ± 5.3	62.7 ± 5.7*
Afro-American	21.2 %	20.3 %	20.5 %	20.3 %	22.0 %	20.3 %	20.3 %	22.0 %	36.2 %	19.2 %*	23.2 %	20.3 %
Female	40.8 %	57.6 %	37.4 %	57.7 %*	35.7 %	56.9 %*	33.9 %	56.9 %*	55.9 %	57.6 %	53.7 %	56.8 %
BMI	29.4 ± 5.7	28.6 ± 5.4	29.5 ± 5.8	28.6 ± 5.4*	28.0 ± 5.8	28.6 ± 5.5	28.1 ± 5.7	28.6 ± 5.5	29.4 ± 5.7	28.6 ± 5.4*	29.9 ± 6.0	28.6 ± 5.4*
Cholesterol (mg/dL)	200.6 ± 34.4	201.4 ± 36.4	200.9 ± 34.2	201.4 ± 36.4	206.9 ± 45.2	201.4 ± 36.3	207.1 ± 44.2	201.4 ± 36.3	200.9 ± 39.1	201.5 ± 36.1	189.4 ± 35.0	201.5 ± 36.3*
Smoking	17.9 %	14.0 %	16.9 %	14.0 %	16.9 %	14.0 %	16.9 %	14.0 %	16.4 %	13.9 %	13.4 %	14.2 %
Hypertension	56.6 %	43.8 %*	54.6 %	44.0 %*	55.4 %	44.4 %	55.9 %	44.4 %	65.1 %	42.6 %*	61.0 %	44.3 %*
Statin Use	10.6 %	9.2 %	9.9 %	9.3 %	17.9 %	9.3 %	17.0 %	9.3 %*	11.3 %	9.1 %	17.1 %	9.2 %*
Diabetes Mellitus	24.1 %	14.3 %*	22.4 %	14.5 %*	35.7 %	14.7 %*	33.9 %	14.7 %*	24.6 %	13.9 %*	23.2 %	14.7 %*

* denotes *p* < 0.05 between those who met criteria and those who did not

**Table 2:** Different ECG Myocardial Infarction Criteria and Risk of Future Coronary Events.

	Standard MC MI		Only Major Q wave abnormality		Standard significant serial Q wave changes		Expanded serial Q wave changes		Standard significant serial Q wave changes or Standard significant serial ST/T changes		Evolving BBB	

SMI Present	3.9 %(*n* = 361)		3.2 % (*n* = 295)		0.6 % (*n* = 54)		0.6 % (*n* = 57)		7.0 % (*n* = 639)		0.8 %(n = 75)	
SMI Rate (Incidence per 1000 patient years; 95 % CI)	4.39 (3.96–4.87)		3.59 (3.20–4.00)		0.66 (0.49–0.0.86)		0.69 (0.52–0.89)		7.78 (7.20–8.37)		0.91 (0.71–1.12)	
	HR (95 % CI)	*p*-value	HR (95 % CI)	p-value	HR (95 % CI)	p-value	HR (95 % CI)	p-value	HR (95 % CI)	p-value	HR (95 % CI)	p-value
Crude	2.54 (1.99–3.18)	<0.001	2.40 (1.84–3.09)	<0.001	4.14 (2.42–6.55)	<0.001	4.71 (2.89–7.21)	<0.001	2.33 (1.92–2.80)	<0.001	1.80 (0.99–2.97)	0.055
Model 1	2.10 (1.65–2.63)	<0.001	1.97 (1.51–2.53)	<0.001	3.30 (1.92–5.22)	<0.001	3.71 (2.27–5.68)	<0.001	2.11 (1.74–2.54)	<0.001	1.49 (0.82–2.46)	0.181
Model 2	1.70 (1.34–2.15)	<0.001	1.65 (1.27–2.14)	<0.001	2.20 (1.33–3.63)	0.002	2.53 (1.60–4.01)	<0.001	1.63 (1.33–1.97)	<0.001	1.39 (0.80–2.40)	0.245

(SMI = Silent Myocardial Infarction; CI = Confidence Interval)

Model 1: Age, Sex, and Race; Model 2: Model 1 +HDL, BMI, Total Cholesterol, Smoking, Systolic Blood Pressure, Antihypertensive Medications, Statin Use, Diabetes Mellitus.

## References

[1] Tsao CW, Aday AW, Almarzooq ZI, Alonso A, Beaton AZ, Bittencourt MS, Boehme AK, Buxton AE, Carson AP, Commodore-Mensah Y, Elkind MSV, Evenson KR, Eze-Nliam C, Ferguson JF, Generoso G, Ho JE, Kalani R, Khan SS, Kissela BM, Knutson KL, Levine DA, Lewis TT, Liu J, Loop MS, Ma J, Mussolino ME, Navaneethan SD, Perak AM, Poudel R, Rezk-Hanna M, Roth GA, Schroeder EB, Shah SH, Thacker EL, VanWagner LB, Virani SS, Voecks JH, Wang NY, Yaffe K, Martin SS. Heart disease and stroke Statistics-2022 update: a report from the American Heart Association. Circulation 2022;145:e153–639. 35078371 10.1161/CIR.0000000000001052

[2] Thygesen K, Alpert JS, Jaffe AS, Simoons ML, Chaitman BR, White HD, Katus HA, Lindahl B, Morrow DA, Clemmensen PM, Johanson P, Hod H, Underwood R, Bax JJ, Bonow RO, Pinto F, Gibbons RJ, Fox KA, Atar D, Newby LK, Galvani M, Hamm CW, Uretsky BF, Steg PG, Wijns W, Bassand JP, Menasché P, Ravkilde J, Ohman EM, Antman EM, Wallentin LC, Armstrong PW, Simoons ML, Januzzi JL, Nieminen MS, Gheorghiade M, Filippatos G, Luepker RV, Fortmann SP, Rosamond WD, Levy D, Wood D, Smith SC, Hu D, Lopez-Sendon JL, Robertson RM, Weaver D, Tendera M, Bove AA, Parkhomenko AN, Vasilieva EJ, Mendis S. Third universal definition of myocardial infarction. Circulation 2012;126:2020–35.22923432

[3] Thygesen K, Alpert JS, Jaffe AS, Chaitman BR, Bax JJ, Morrow DA, White HD. Fourth universal definition of myocardial infarction (2018). Circulation 2018;138:e618–51. 30571511 10.1161/CIR.0000000000000617

[4] Luepker RV, Apple FS, Christenson RH, Crow RS, Fortmann SP, Goff D, Goldberg RJ, Hand MM, Jaffe AS, Julian DG, Levy D, Manolio T, Mendis S, Mensah G, Pajak A, Prineas RJ, Reddy KS, Roger VL, Rosamond WD, Shahar E, Sharrett AR, Sorlie P, Tunstall-Pedoe H. Case definitions for acute coronary heart disease in epidemiology and clinical research studies: a statement from the AHA Council on epidemiology and prevention; AHA statistics committee; world heart federation council on epidemiology and prevention; the European Society of Cardiology Working Group on epidemiology and prevention; Centers for Disease Control and Prevention; and the National Heart, Lung, and Blood Institute. Circulation 2003;108:2543–9. 14610011 10.1161/01.CIR.0000100560.46946.EA

[5] Gibson CM, Nafee T, Kerneis M. Silent myocardial infarction: listen to the evidence. J Am Coll Cardiol 2018;71:9–11. 29301632 10.1016/j.jacc.2017.10.069

[6] Margolis JR, Kannel WS, Feinleib M, Dawber TR, McNamara PM. Clinical features of unrecognized myocardial infarction–silent and symptomatic. Eighteen year follow-up: the Framingham study. Am J Cardiol 1973;32:1–7. 4713110 10.1016/s0002-9149(73)80079-7

[7] Zhang ZM, Rautaharju PM, Prineas RJ, Rodriguez CJ, Loehr L, Rosamond WD, Kitzman D, Couper D, Soliman EZ. Race and sex differences in the incidence and prognostic significance of silent myocardial infarction in the atherosclerosis risk in communities (ARIC) study. Circulation 2016;133:2141–8. 27185168 10.1161/CIRCULATIONAHA.115.021177 PMC4889519

[8] Pride YB, Piccirillo BJ, Gibson CM. Prevalence, consequences, and implications for clinical trials of unrecognized myocardial infarction. Am J Cardiol 2013;111:914–8. 23276472 10.1016/j.amjcard.2012.11.042

[9] Sheifer SE, Gersh BJ, Yanez ND 3rd, Ades PA, Burke GL, Manolio TA. Prevalence, predisposing factors, and prognosis of clinically unrecognized myocardial infarction in the elderly. J Am Coll Cardiol 2000;35:119–26. 10636269 10.1016/s0735-1097(99)00524-0

[10] Valensi P, Lorgis L, Cottin Y. Prevalence, incidence, predictive factors and prognosis of silent myocardial infarction: a review of the literature. Arch Cardiovasc Dis 2011;104:178–88. 21497307 10.1016/j.acvd.2010.11.013

[11] Kannel WB, Abbott RD. Incidence and prognosis of unrecognized myocardial infarction. An update on the Framingham study. N Engl J Med 1984;311:1144–7. 6482932 10.1056/NEJM198411013111802

[12] Nadelmann J, Frishman WH, Ooi WL, Tepper D, Greenberg S, Guzik H, Lazar EJ, Heiman M, Aronson M. Prevalence, incidence and prognosis of recognized and unrecognized myocardial infarction in persons aged 75 years or older: the Bronx aging study. Am J Cardiol 1990;66:533–7. 2392974 10.1016/0002-9149(90)90477-i

[13] Boland LL, Folsom AR, Sorlie PD, Taylor HA, Rosamond WD, Chambless LE, Cooper LS. Occurrence of unrecognized myocardial infarction in subjects aged 45 to 65 years (the ARIC study). Am J Cardiol 2002;90:927–31. 12398956 10.1016/s0002-9149(02)02655-3

[14] Qureshi WT, Zhang ZM, Chang PP, Rosamond WD, Kitzman DW, Wagenknecht LE, Soliman EZ. Silent myocardial infarction and long-term risk of heart failure: the ARIC study. J Am Coll Cardiol 2018;71:1–8. 29301615 10.1016/j.jacc.2017.10.071 PMC5757248

[15] Merkler AE, Bartz TM, Kamel H, Soliman EZ, Howard V, Psaty BM, Okin PM, Safford MM, Elkind MSV, Longstreth WT Jr. Silent myocardial infarction and subsequent ischemic stroke in the cardiovascular health study. Neurology 2021;97:e436–43. 34031202 10.1212/WNL.0000000000012249 PMC8356380

[16] Crow RS, Prineas RJ, Hannan PJ, Grandits G, Blackburn H. Prognostic associations of Minnesota code serial electrocardiographic change classification with coronary heart disease mortality in the multiple risk factor intervention trial. Am J Cardiol 1997;80:138–44. 9230148 10.1016/s0002-9149(97)00307-x

[17] Vähätalo JH, Huikuri HV, Holmström LTA, Kenttä TV, Haukilahti MAE, Pakanen L, Kaikkonen KS, Tikkanen J, Perkiömäki JS, Myerburg RJ, Junttila MJ. Association of Silent Myocardial Infarction and Sudden Cardiac Death. JAMA Cardiol 2019;4:796–802. 31290935 10.1001/jamacardio.2019.2210 PMC6624824

[18] Leening MJ, Elias-Smale SE, Felix JF, Kors JA, Deckers JW, Hofman A, Stricker BH, Witteman JC. Unrecognised myocardial infarction and long-term risk of heart failure in the elderly: the Rotterdam study. Heart (British Cardiac Society) 2010;96:1458–62. 20483894 10.1136/hrt.2009.191742

[19] Davis TM, Coleman RL, Holman RR. Prognostic significance of silent myocardial infarction in newly diagnosed type 2 diabetes mellitus: United Kingdom prospective diabetes study (UKPDS) 79. Circulation 2013;127:980–7. 23362315 10.1161/CIRCULATIONAHA.112.000908

[20] Zhang ZM, Prineas RJ, Soliman EZ, Baggett C, Heiss G. Prognostic significance of serial Q/ST-T changes by the Minnesota code and Novacode in the atherosclerosis risk in communities (ARIC) study. Eur J Prev Cardiol 2012;19:1430–6. 21997257 10.1177/1741826711426091 PMC4948579

[21] Rautaharju PM, Prineas RJ, Wood J, Zhang ZM, Crow R, Heiss G. Electrocardiographic predictors of new-onset heart failure in men and in women free of coronary heart disease (from the atherosclerosis in communities [ARIC] study). Am J Cardiol 2007;100:1437–41. 17950804 10.1016/j.amjcard.2007.06.036

[22] Kannel WB. Prevalence and clinical aspects of unrecognized myocardial infarction and sudden unexpected death. Circulation 1987;75. Ii4–5. 3493089

[23] Soliman EZ. Electrocardiographic definition of silent myocardial infarction in population studies: a call to standardize the standards. J Electrocardiol 2019;55:128–32. 31170595 10.1016/j.jelectrocard.2019.05.008

[24] Levitan EB, Safford MM, Kilgore ML, Soliman EZ, Glasser SP, Judd SE, Muntner P. Assessment tools for unrecognized myocardial infarction: a cross-sectional analysis of the REasons for geographic and racial differences in stroke population. BMC Cardiovasc Disord 2013;13:23. 23530553 10.1186/1471-2261-13-23 PMC3617994

[25] Ammar KA, Kors JA, Yawn BP, Rodeheffer RJ. Defining unrecognized myocardial infarction: a call for standardized electrocardiographic diagnostic criteria. Am Heart J 2004;148:277–84. 15308997 10.1016/j.ahj.2004.03.019

[26] Ammar KA, Samee S, Makwana R, Urban L, Mahoney DW, Kors JA, Redfield MM, Jacobsen S, Rodeheffer RJ. Echocardiographic characteristics of electrocardiographically unrecognized myocardial infarctions in a community population. Am J Cardiol 2005;96:1069–75. 16214440 10.1016/j.amjcard.2005.06.036

[27] Kwong RY, Chan AK, Brown KA, Chan CW, Reynolds HG, Tsang S, Davis RB. Impact of unrecognized myocardial scar detected by cardiac magnetic resonance imaging on event-free survival in patients presenting with signs or symptoms of coronary artery disease. Circulation 2006;113:2733–43. 16754804 10.1161/CIRCULATIONAHA.105.570648

[28] Wright JD, Folsom AR, Coresh J, Sharrett AR, Couper D, Wagenknecht LE, Mosley TH Jr, Ballantyne CM, Boerwinkle EA, Rosamond WD, Heiss G. The ARIC (atherosclerosis risk in communities) study: JACC focus seminar 3/8. J Am Coll Cardiol 2021 Jun 15;77(23):2939–59. 34112321 10.1016/j.jacc.2021.04.035 PMC8667593

[29] Prineas RJ, Crow RS, Zhang Z-M. The Minnesota code manual of electrocardiographic findings. London: Springer London; 2010.

[30] White AD, Folsom AR, Chambless LE, Sharret AR, Yang K, Conwill D, Higgins M, Williams OD, Tyroler HA. Community surveillance of coronary heart disease in the atherosclerosis risk in communities (ARIC) study: methods and initial two years’ experience. J Clin Epidemiol 1996;49:223–33. 8606324 10.1016/0895-4356(95)00041-0

[31] Rosamond WD, Chambless LE, Heiss G, Mosley TH, Coresh J, Whitsel E, Wagenknecht L, Ni H, Folsom AR. Twenty-two-year trends in incidence of myocardial infarction, coronary heart disease mortality, and case fatality in 4 US communities, 1987–2008. Circulation 2012;125:1848–57. 22420957 10.1161/CIRCULATIONAHA.111.047480 PMC3341729

[32] Conti CR, Bavry AA, Petersen JW. Silent ischemia: clinical relevance. J Am Coll Cardiol 2012;59:435–41. 22281245 10.1016/j.jacc.2011.07.050

[33] Gupta M, Miller CJ, Baker JV, Lazar J, Bogner JR, Calmy A, Soliman EZ, Neaton JD. Biomarkers and electrocardiographic evidence of myocardial ischemia in patients with human immunodeficiency virus infection. Am J Cardiol 2013;111:760–4. 23276469 10.1016/j.amjcard.2012.11.032 PMC3619189

[34] Dehghan A, Leening MJ, Solouki AM, Boersma E, Deckers JW, van Herpen G, Heeringa J, Hofman A, Kors JA, Franco OH, Ikram MA, Witteman JC. Comparison of prognosis in unrecognized versus recognized myocardial infarction in men versus women >55 years of age (from the Rotterdam study). Am J Cardiol 2014;113:1–6. 24216125 10.1016/j.amjcard.2013.09.005

[35] Michael MA, El Masry H, Khan BR, Das MK. Electrocardiographic signs of remote myocardial infarction. Prog Cardiovasc Dis 2007;50:198–208. 17976504 10.1016/j.pcad.2007.05.003

[36] Turkbey EB, Nacif MS, Guo M, McClelland RL, Teixeira PB, Bild DE, Barr RG, Shea S, Post W, Burke G, Budoff MJ, Folsom AR, Liu CY, Lima JA, Bluemke DA. Prevalence and correlates of myocardial scar in a US cohort. Jama 2015;314:1945–54. 26547466 10.1001/jama.2015.14849 PMC4774246

